# Parental Engagement of a Prototype Electronic Diary in an Ambulatory Setting Following Adenotonsillectomy in Children: A Prospective Cohort Study

**DOI:** 10.3390/children8070559

**Published:** 2021-06-29

**Authors:** Tobial Mchugh, Karen A. Brown, Sam J. Daniel, Sharmila Balram, Chantal Frigon

**Affiliations:** 1 Department of Otorhinolaryngology, McGill University Health Center, Montreal Children’s Hospital, Montréal, QC H4H 3J1, Canada; tobial.mchugh@medportal.ca (T.M.); sam.j.daniel@mcgill.ca (S.J.D.); 2 Department of Anesthesiology, McGill University Health Center, Montreal Children’s Hospital, Montréal, QC H4H 3J1, Canada; karen.brown@mcgill.ca (K.A.B.); sharmila.balram@affiliate.mcgill.ca (S.B.)

**Keywords:** children, pain measurement, electronic pain diary, pain scale, PPPM, parent’s postoperative pain measure, adenotonsillectomy

## Abstract

Adenotonsillectomy is performed in children on an outpatient basis, and pain is managed by parents. A pain diary would facilitate pain management in the ambulatory setting. Our objective was to evaluate the parental response rate and the compliance of a prototype electronic pain diary (e-diary) with cloud storage in children aged 2–12 years recovering from adenotonsillectomy and to compare the e-diary with a paper diary (p-diary). Parents recorded pain scores twice daily in a pain diary for 2 weeks post-operation. Parents were given the choice of an e-diary or p-diary with picture message. A total of 208 patients were recruited, of which 35 parents (16.8%) chose the e-diary. Most parents (98%) chose to be contacted by text message. Eighty-one families (47%) returned p-diaries to us by mail. However, the response rate increased to 77% and was similar to that of the e-diary (80%) when we included data texted to the research phone from 53 families. The proportion of diaries with *Complete* (e-diary:0.37 vs. p-diary:0.4) and *Incomplete* (e-diary:0.43 vs. p-diary:0.38) data entries were similar. E-diaries provide a means to follow patients in real time after discharge. Our findings suggest that a smartphone-based medical health application coupled with a cloud would meet the needs of families and health care providers alike.

## 1. Introduction

Adenotonsillectomy is one of the most common surgeries performed in the pediatric population, and the vast majority of children are discharged from hospital the same day. The burden of managing postoperative pain at home is delegated to the primary caregiver. However, despite perioperative education emphasizing expectations and discharge instructions, there is evidence that for many of these children pain persists for 2 weeks following TA and that the pain is not adequately controlled [1,2,3,4].

Pain assessment in children requires that parents repeatedly evaluate pain throughout the day and night. Common tools to facilitate pain assessment in the outpatient setting are a pain scale and a pain diary. When completed prospectively, there is evidence that the use of pain diaries increased the overall validity of children’s pain reports compared with retrospective interviews [5] and improved accuracy [6]. Historically, paper diaries were employed and data were entered manually by the parents. The disadvantages of paper diaries include the incomplete records and non-adherence to or deviation from the protocol. Moreover one cannot assume that a parent did not enter all data in a single day [6]. Furthermore, paper diaries are known to have a relatively low response rate, ranging from 11% to 40% [6,7].

Electronic diaries are amenable to cloud storage. Cloud storage is a relatively inexpensive storage platform and provides a solution for real-time oversight and the analysis of time series patient information by health care providers [8]. Thus, we created a web based prototype electronic pain diary (e-diary) that included the cloud storage of anonymized data.

The primary aim was to evaluate parental engagement with a prototype electronic pain diary (e-diary) in a population of children recovering from adenotonsillectomy and to compare it with that of a paper diary (p-diary). We defined parental engagement as the diary response rate and the compliance for diary usage. A secondary aim was to determine the parental satisfaction with the e-diary.

## 2. Materials and Methods

### 2.1. Study Design and Setting

**Figure 1:**
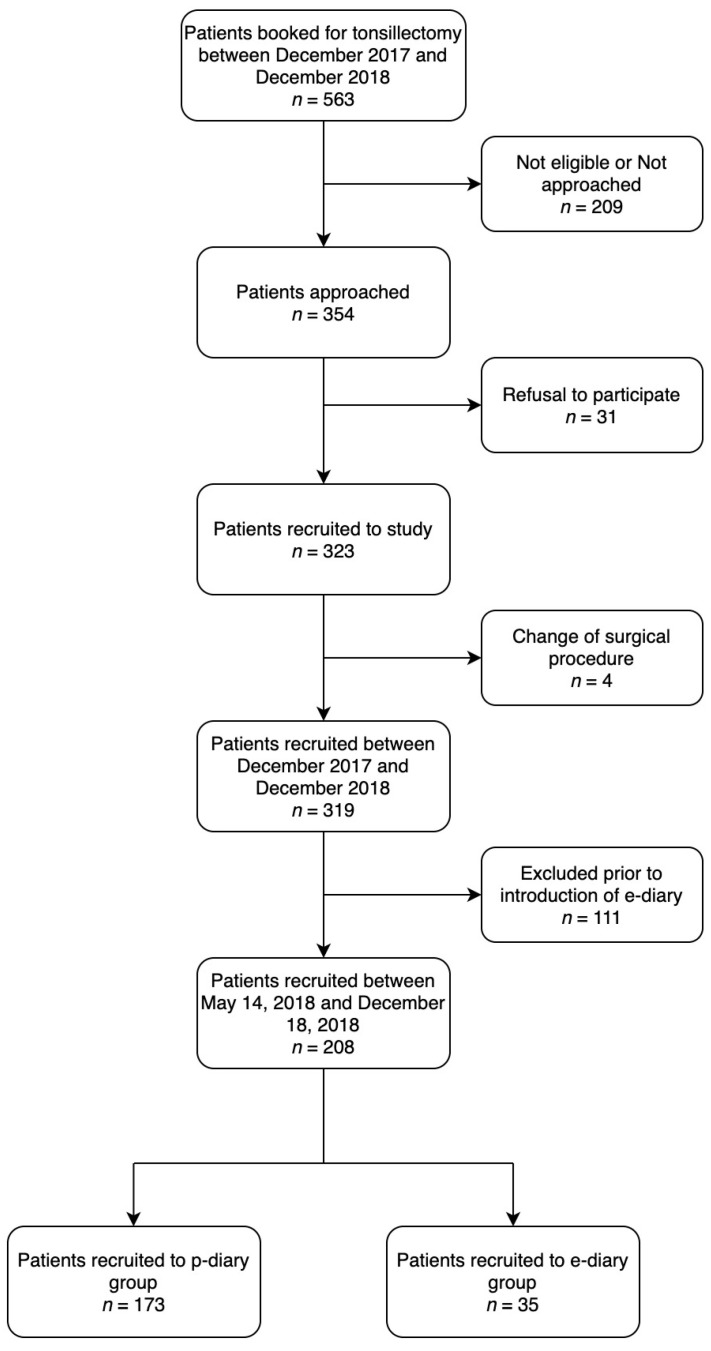
Flowchart recruitment. Legend: p-diary, paper pain diary; e-diary, electronic pain diary.

The current study represents a subset of children recruited to a prospective cohort study to assess pain management at home after an adenotonsillectomy conducted at the Montreal Children’s Hospital, Quebec, Canada from 19 December 2017 to 18 December 2018. Between 14 May and 18 December 2018, parents were given the choice of an e-diary or a p-diary. (Figure 1) The study was reviewed and approved by the McGill University Health Center (MUHC) Research Ethics Board (Approval number 2017-3127) and was registered with Clinical Trials gov ID #NCT03378830.

Parents were informed of the research study in advance of surgery, and informed written parental consent and child assent when appropriate was obtained on the day of surgery. Patients were assigned a unique alphanumeric identifier.

On the day of the surgery, the research nurse taught parents how to use two age-appropriate pain scales; namely the Parents’ Postoperative Pain Measure (PPPM) [9] plus a second scale: the Face, Legs, Activity, Cry and Consolability (FLACC) [10] pain scale for children 2–3 years old or the Faces Pain Scale—Revised (FPS-R) [11] for children aged 4 years and older.

Parents were asked to record the pain scores of their child twice daily, morning and evening, for 2 weeks following surgery and to record the analgesic medications given daily. They were also asked to comment on the reasons underlying their choice of diary types. The e-diary had two embedded educational videos: (1) *How to complete the e–diary* and (Video S1) (2) *Instructions for postoperative care* (Video S2).

In both diary groups, the research nurse contacted the parents by text message or by phone on postoperative days (POD) 3, 6, 9, and 12 via the research cell phone. The alphanumeric identifier was used to register the child with the application ASANA (Asana Inc., version 6.8.0, 2018, San Francisco, CA, USA) to schedule these contacts. During this contact, the pain scores and administration of medication were reviewed. In order to reduce the burden on parents, they were told that they could stop entering data if the PPPM scores were zero for two consecutive PODs.

### 2.2. Participants

We used consecutive sampling of children who were recruited to the aforementioned study. Children between the ages of 2 and 12 years undergoing elective tonsillectomy ± adenotonsillectomy (TA) were eligible for recruitment. Inclusion criteria were the American Society of Anesthesiologists (ASA) physical status [12] of 1, 2, or 3, and fluency in either English or French. Exclusion criteria were: (i) medical complexity including neuromuscular disease, seizure disorders, cyanotic heart disease, Trisomy 21, craniofacial syndromes, steroid dependant asthma, cystic fibrosis, severe autism spectrum disorder, and (ii) moderate to severe developmental delay, cognitive or neurological conditions which might impair the assessment. We did not ask parents the reason for declining participation in the study, but several parents told us that they would not have time to meet the study’s requirement.

### 2.3. Data Collection

**Figure 2:**
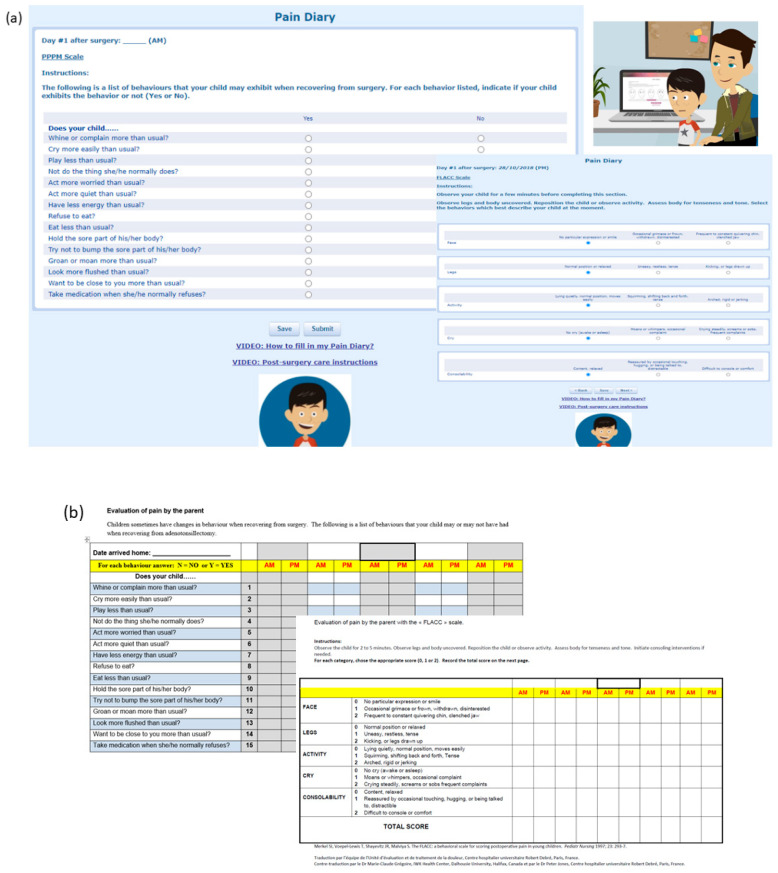
Representative illustrations of PPPM and FLACC diary entries for the electronic (**a**) and for the paper (**b**) diaries. Legend: PPPM, Parent’s Postoperative Pain Measurement; FLACC, Faces, Legs, Activity, Cry, Consolability Pain scale.

Representative examples of the diaries are shown in Figure 2. The p-diary was given to the families on the day of surgery. Parents were asked to picture-message the data entries to the research phoneon POD3, 6, 9, and 12 and to mail the completed paper diary to us. Parents who chose the e-diary were asked to enter data directly online with a link provided to a webpage designed for this project by a company called the AXDEV group (AXDEV Group, Brossard, QC, Canada). The AXDEV’s online platform had many advantages in terms of infrastructure and data security. E-diary data entries were recorded and stored in a secure state-of-the-art SSAE-16 compliant facility on a platform that complied with ISO 27001 standard for information security. Thus, the research team was able to log into the webpage and follow, in real-time, the evolution of the pain scores and medication administration. Upon completion of the e-diary, parents were sent a satisfaction survey (Appendix A).

### 2.4. Data Sources and Measures

There were two sources of data: the medical dossier and the pain diaries.

#### 2.4.1. Pain

The PPPM is an observational, 15-item behavioral scale which has been validated for use in the postoperative home setting [9]. The FLACC pain scale is a 10-point behavioral scale for quantifying pain in children too young to use a self-report pain scale. It is validated for children less than 4 years of age [10,11]. The FPS-R is a 10-point metric self-report pain scale validated for children 4–12 years of age [11].

#### 2.4.2. Parental Engagement (Response Rate and Compliance)

The diary response rate was defined as the number of participants who submitted any data from the pain diary during the recovery period divided by the total number of participants.

Compliance with diary usage was classified as *Complete*, *Incomplete*, and *No Data* and reported as *n* (%). There were four types of diary entries: (1) pain data entries on POD 14, (2) pain data entries until the parents were told to stop (Told-to-Stop), (3) incomplete pain data entries, and (4) lost to follow-up. We combined diary entry classes (1) and (2) as a single group: *Complete*. The label *Complete* was assigned even if there were missing data or time points between POD1 and POD14 if data for POD14 were entered. *Incomplete* data entries occurred when parents completed entries for at least one POD but dropped out before POD14. *No Data* refers to parents who were lost to follow-up from whom no data were submitted.

Last POD entry for medication, PPPM score, and second pain score were reported.

#### 2.4.3. Satisfaction Ratings

For parents who completed the e-diary, the satisfaction questionnaire presented questions regarding the ease of connection to the webpage, the transition time between questions, the clarity of instructions, and the overall satisfaction. There were also questions related to the two videos embedded in the diary. (See Supplementary Materials, Videos S1 and S2).

### 2.5. Data Analysis and Statistical Analysis

Clinicodemographic data were summarized using descriptive statistics. The diary response rate and compliance were reported as *n* (%). Clinicodemographic characteristics according to diary group (p-diary vs. e-diary) were analyzed with Chi-square or Fisher’s exact tests for categorical variables. As continuous variables were not normally distributed, they are presented as median and interquartile range (IQR) and were analyzed with the Wilcoxon rank sum test. Statistical analysis was performed using *R Foundation* for Statistical Computing (2019), version 3.6.2, Vienna, Austria. URL https://www.R-project.org/, (accessed on 2 March 2021). A *p*-value of < 0.05 was considered significant.

## 3. Results

### 3.1. Demographic and Clinical Characteristics

Overall, 208 patients were recruited during the period when both diaries were offered: p-diaries (173), e-diaries (35). The median age and weight were similar between the p- and e-diary groups (Table 1). In both groups, most of the teaching on the pain scales and the diary was provided to the patient’s mother (65.7%). Most parents (97%) chose to be contacted by the research nurse by text message instead of a phone call. The majority (83%) of parents chose the p-diary (with picture message); 35 parents (16.8%) chose the e-diary. Reasons for these choices included:

- “I do not have time to sit at computer”
- “I will not be able to look back at the progression”
- “I will not have time (I am home alone)”
- “It would be easier to send the photos (picture message)”
- “Had the e-diary been available on a cell phone APP, I would have done electronic.”

### 3.2. Parental Engagement of the Prototype E-Diary

#### 3.2.1. Diary Response Rate

Overall, the diary response rate was 78% (162/208). In total there were 77 complete, 80 incomplete, 5 Told-to-Stop, and 46 lost to follow-up diaries. The median POD with the last data entry for incomplete diaries was 8 [IQR 4, 10] and for Told-to-Stop 10 [IQR 10, 12].

Eighty-one families (47%) returned p-diaries to us by mail; we obtained images of data texted to the research phone from 53 additional families. Thus, the response rate for the p-diary was 77%, comparable to the 80% for the e-diary.

#### 3.2.2. Compliance with Diary Usage

**Figure 3:**
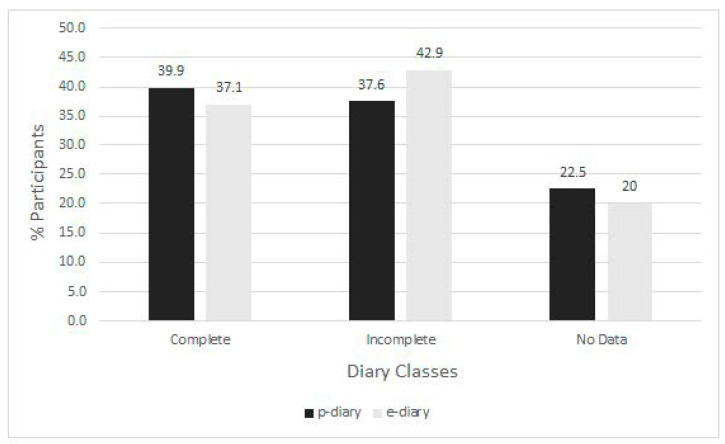
Compliance with daily diary recordings among the paper (black) and electronic (grey) pain diaries. Legend: p-diary, paper pain diary; e-diary, electronic pain diary. See text for definition of Diary Classes.

Compliance with the p- and e-diary classes were similar. (Figure 3)

In both groups, less than 6% of diaries had gaps for PPPM and 2nd pain scale (Table 2). PPPM items were missing more frequently in the p-diary group compared to the e-group (14 (10.4%) vs. 1 (3.6%), respectively), but this difference was not statistically significant.

For the e-diary, the median POD for last data entry for the PPPM scale was POD13 [IQR 4.75, 14] for Complete and POD 5 [IQR 3, 10] for Incomplete. For the p-diary, the median POD for last data entry for PPPM was POD12 [IQR 8, 14] for Complete and POD 8 [IQR 5, 10] for Incomplete (*p* = 0.654).

### 3.3. Parental Satisfaction with the Prototype E-Diary

Fourteen parents (40.0%) completed the satisfaction questionnaire for the e-diary (Figure S1), and 93% of parents were in total agreement or agreement with the fact that the instructions were clear and easy to understand, while only 29% of parents were very satisfied with the transition time between each question, and 21% found that the diary took more than a little of their time to fill daily (Figure S1). Three parents commented that the transition time between questions was too long, which made the e-diary annoying to complete. Despite the dissatisfaction regarding the speed of the e-diary, the overall satisfaction was excellent; 86% of parents were either satisfied or very satisfied with the overall experience. Parents commented that the e-diary was a useful tool to follow the evolution of their child’s pain after the surgery.

Regarding the e-diary’s embedded videos, the majority of patients (85.7%) found that the two videos on how to fill out the pain diary and on how to take care of a child after TA were useful and that their content was relevant.

## 4. Discussion

A means to monitor patients during the postoperative period in the ambulatory setting is desirable to health care providers. Indeed, 77 families submitted complete diaries with data for the 14 POD. The median POD for last data entry for incomplete diaries was POD8 [IQR 4, 10] and for Told-to-Stop POD10 [IQR 10, 12], suggesting that an additional 45 families (cumulative total 75%) also found the diaries useful. Comments from parents identified the e-diary as a useful tool to follow the evolution of their child’s pain following adenotonsillectomy. Overall, 86% of parents were either satisfied or very satisfied with the prototype e-diary.

Given the widespread use of information technology in society, we were surprised that a minority of parents (16.8%) chose the e-diary. Parents indicated that the ease of use was a major factor influencing this choice. Whereas web-based platforms are attractive to health care providers, applications (APPs) for mobile devices provide a more appealing interface for families. Indeed, most parents preferred the convenience of handheld mobile devices. Firstly, an overwhelming majority of parents (97%) chose to be contacted via text message to their cell phones. In addition, parents preferred to write the pain scores on paper and communicate the data via picture text message to the research cellphone. Indeed, some parents reported that the requirement to log onto a web site and enter data electronically made the e-diary unattractive.

Cloud storage of data allowed both the parents and research team to follow the evolution of pain while the child recovered at home. The research team followed the pain scores daily with the e-diary. In contrast, the pain scores in the p-diary could only be reviewed every 3 days, following receipt of the picture images. In our study, text messages via the research phone also allowed a two-way communication between the research nurse and the families. However, this was more labor intensive than cloud storage, as it required contacts with the families by the research team.

We had anticipated a higher parental engagement within the e-diary group, but the response rate and compliance for both diaries were similar (Figure 3). In contrast, Palermo et al. [7] provided an e-diary with a personal digital assistant and reported a higher compliance with the e-diary compared to a p-diary in a population of adolescents with chronic pain (83.3% vs. 46.7%, respectively). Stone et al. [6] reported a significantly higher (94% vs. 11%) compliance with e-diaries compared to p-diaries in a study of adults with chronic pain, attributing this result to electronic reminders and alarms. These additional automated features in e-diaries may improve diary completion rates and suggest an advantage of the electronic platform for future work.

It became clear as the study progressed that the communication platform was a critical factor in the choice between the p- and e-diaries. For example, text message was a feature embraced by families who chose the p-diary. The scheduled scripted contacts to families on POD3, 6, 9, and 12 allowed us to retrieve data from an additional 53 families who did not mail their p-diaries to the research team. Smartphone mobile health applications provide patient-centered care by facilitating follow-up during the postoperative period [13]. Chen et al. [4] reported a response rate of 88% using a short message service (SMS) to assess post-tonsillectomy pain at home where scheduled and automated messages were sent daily asking parents to message back the child’s pain score.

When we looked at the *Incomplete* diaries (*n* = 80), participants with the e-diary tended to drop out 3 days earlier (POD5 [IQR 3, 10]) than participants with the p-diary (POD8 [IQR 5, 10]). Dissatisfaction with the slow transition time may have contributed to the earlier dropout for the e-diary. A parent from the e-diary group commented that “It was annoying to do, due to the amount of wait time (transition delay) between each question”.

The e-diary mitigated missing items because parents could not transition before all items were scored. This was an advantage for data collection although it may have irritated parents. Another advantage of the e-diary is that it provided a time stamp (date and time) of the data entry, thereby mitigating the errors inherent in p-diaries which arise from the retrospective recollection of events and details [6,14].

It is perceived that perioperative education will mitigate the high rates of visits to the emergency room and readmissions following adenotonsillectomy. Thus, we felt it was advantageous to embed two educational videos in the e-diary, providing parents with accessible information without the need to log onto another website or locate paper documents. The second video provided information on postoperative care after tonsillectomy. Parents indicated that these videos were useful.

This study has limitations. Parents were given the choice of which type of diary they would use. We did not randomize the diary type, as we wanted to maximize parents’ adhesion to the protocol of the initial study on pain assessment after TA for the 14 days period. The satisfaction survey was not sent to parents with the p-diary; reflecting our focus for this study on the prototype e-diary. A type II error may have occurred as the sample size of the e-diary group was small.

The impact of educational material on other key performance indicators is not clear, as there are conflicting results. Levin et al. [15] reported that pre-tonsillectomy educational programs including smartphone applications were effective in decreasing visits to the emergency department, patient anxiety, as well as improved pain management. In contrast, Jain et al. [16] did not find a difference when comparing three different methods of perioperative education. Educational videos did not improve parent satisfaction compared to more conventional education consisting of verbal counseling and leaflets [17]. An Internet-based method improved the acquisition of knowledge and satisfaction of adolescents and their parents, but did not reduce patient or parental anxiety compared to traditional education and even the absence of preoperative education [18]. Interestingly, the education material provided by text messages both increased the knowledge of mothers and decreased anxiety following tonsillectomy [19,20].

## 5. Conclusions

This study sought feedback from parents to inform the development of a prototype e-diary with cloud storage suitable for use in the home environment. We concluded that e-diaries provided a means to follow patients after discharge from hospital. The reasons for parents to decline the e-diary should be considered for the future version of the e-diary, and a satisfaction questionnaire should be administered to both diary groups. The preference to communicate with text message and picture images suggests that an e-diary configured as an application for cell phones would be desirable for parents. Future work needs to explore communication platforms that both ensure anonymity and ease of use.

## Figures and Tables

**Table 1:** Clinico-demographic Characteristics between the Paper Diary and Electronic Diary Groups.

	Paper Diary (*n* = 173)	Electronic Diary (*n* = 35)	*p*-Value
	**Median [IQR]**	**Median [IQR]**	
**Age (years)**	4.3 [3.0–6.0]	3.9 [3.2–4.9]	0.296
**Weight (kg)**	17.6 [14.9–24.1]	16.9 [14.7–22.2]	0.293
	***n* (%)**	***n* (%)**	
**Gender = Female**	75 (43.3)	17 (48.6)	0.135
**ASA**			
1	91 (52.6)	16 (45.7)	0.612
2	78 (45.1)	18 (51.4)	
3	4 (2.3)	1 (2.9)	
4	0 (0.0)	0 (0.0)	
**Surgical Indications**			
OSA	55 (31.8)	14 (40.0)	0.936
TAH	90 (52.0)	16 (45.7)	
Recurrent Tonsillitis	15 (8.7)	1 (2.9)	
Undetermined	13 (7.5)	4 (11.4)	

Legend: *n*, count; IQR, Interquartile range; ASA, American Society of Anesthesiologists physical status; OSA, Obstructive Sleep Apnea; TAH, Tonsil Adenoid Hypertrophy.

**Table 2:** Features of the Paper Diary and Electronic Diary groups.

	Paper Diary (*n* = 173)	Electronic Diary (*n* = 35)	*p*-Value
	***n* (%)**	***n* (%)**	
**Teaching given to:**			
Mother	113 (65.7)	23 (67.6)	0.150
Father	9 (5.2)	3 (8.8)	
Both	50 (29.1)	7 (20.6)	
Other	0 (0.0)	1 (2.9)	
**Diaries with pain entries (excludes entries with no data)**	(*n* = 134)	(*n* = 28)	
Gaps for PPPM	8 (6.0)	1 (3.6)	1.000
Gaps for 2nd Pain Scale	5 (3.7)	0 (0.0)	0.589
Missing PPPM items	14 (10.4)	1 (3.6)	0.472
	**Median [IQR]**	**Median [IQR]**	
**Last POD entry for:**			
Medication	13 [7.00–14.00]	13 [3.75–14.00]	0.554
PPPM score	12 [8.00–14.00]	13 [4.75–14.00]	0.654
2nd Pain Scale score	13 [8.25–14.00]	13 [4.75–14.00]	0.526

Legend: IQR, Interquartile range; POD, postoperative day; PPPM, Parent’s Postoperative Pain Measure; 2nd Pain Scale, Faces, Legs, Activity, Cry, Consolability Pain Assessment Tool (FLACC) for children 2–3 years old or Face Pain Scale-Revised (FPS-R) for children 4–12 years old.

## Data Availability

Data are available upon request.

## Supplementary Materials

The following are available online at https://www.mdpi.com/article/10.3390/children8070559/s1, Figure S1: Parents’ responses to the first 6 questions of the satisfaction questionnaire for the participants who completed the e-diary (*n* = 14), Video S1: How to complete the e-diary, https://youtu.be/29dS0AhBwb8, (accessed on 28 June 2021). Video S2: Instructions for postoperative care, https://youtu.be/YXZ07Z4y4JM, (accessed on 28 June 2021).

## Appendix A

Satisfaction questionnaire provided to the electronic diary group:

**Q1: Please indicate your satisfaction with the ease of connection.** 

Answer Choices

- Very satisfied
- Satisfied
- Neither satisfied nor dissatisfied
- Dissatisfied
- Very dissatisfied

**Q2. Please indicate how satisfied were you with the transition time between questions.**

Answer Choices

- Very satisfied
- Satisfied
- Neither satisfied nor dissatisfied
- Dissatisfied
- Very dissatisfied

**Q3. The instructions were clear and easy to understand.**

Answer Choices

- In total agreement
- Agree
- Neither agree nor disagree
- Disagree
- In total disagreement

**Q4. The journal took up little of my time to fill daily.**

Answer Choices

- In total agreement
- Agree
- Neither agree nor disagree
- Disagree
- In total disagreement

**Q5. Please indicate how satisfied were you with your overall experience with the electronic pain diary.**

Answer Choices

- Very satisfied
- Satisfied
- Neither satisfied nor dissatisfied
- Dissatisfied
- Very dissatisfied

**Q6. Would you recommend this diary to other families?**

Answer Choices

- Yes
- No
- Don’t know

**Q7. Please indicate your level of agreement with the following statements about the video “How to fill out my Pain Diary?”: The content was relevant.**

Answer Choices

- In total agreement
- Agree
- Neither agree nor disagree
- Disagree
- In total disagreement

**Q8. Please indicate your level of agreement with the following statements about the video “How to fill out my Pain Diary?”: In general, the video was useful to me.**

Answer Choices

- In total agreement
- Agree
- Neither agree nor disagree
- Disagree
- In total disagreement

**Q9. Please indicate your level of agreement with the following statements about the video “Postoperative care instructions?”: The content was relevant.**

Answer Choices

- In total agreement
- Agree
- Neither agree nor disagree
- Disagree
- In total disagreement

**Q10. Please indicate your level of agreement with the following statements about the video “Postoperative care instructions”: In general, the video was useful to me.**

Answer Choices

- In total agreement
- Agree
- Neither agree nor disagree
- Disagree
- Inn total disagreement
