# Supplementary material for: Parental Engagement of a Prototype Electronic Diary in an Ambulatory Setting Following Adenotonsillectomy in Children: A Prospective Cohort Study
Source: Children (Basel). 2021 Jun 29;8(7):559. doi: 10.3390/children8070559 (PMC8303765; doi:10.3390/children8070559)
Supplement: Supplementary file 1 [file children-08-00559-s001.zip › children-1237843-supplemeantary.pdf]

children

Supplementary Material:

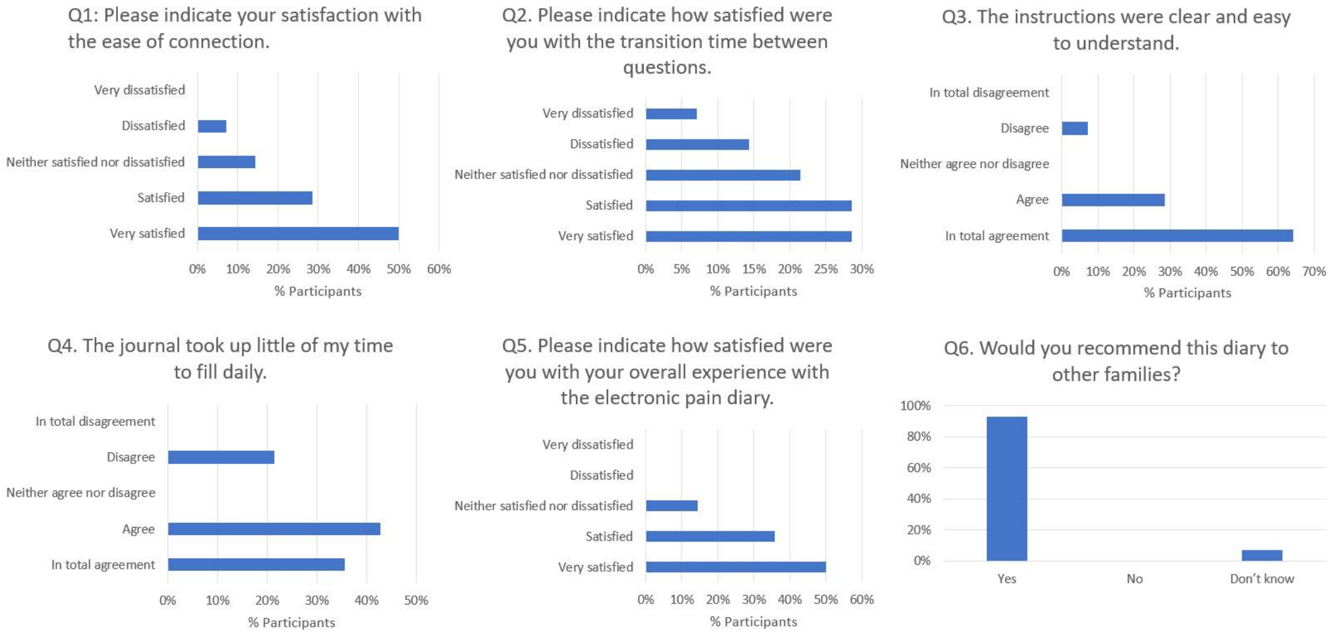

Figure S1. Parents' responses to the first 6 questions of the satisfaction questionnaire for the participants who completed the e-diary (n = 14).
